# Assessing public behavioral health services data: a mixed method analysis

**DOI:** 10.1186/s13011-020-00328-9

**Published:** 2020-11-11

**Authors:** Sierra X. Vaughn, Hannah L. Maxey, Alyson Keen, Kelli Thoele, Robin Newhouse

**Affiliations:** 1 Indiana University School of Medicine, 1110 West Michigan St., Suite 200, Indianapolis, Indiana 46202 USA; 2 Indiana University School of Nursing, 600 Barnhill Dr., Indianapolis, Indiana 46202 USA

**Keywords:** Substance abuse treatment centers, Health services accessibility, Public reporting of healthcare data

## Abstract

**Background:**

Measuring behavioral health treatment accessibility requires timely, comprehensive and accurate data collection. Existing public sources of data have inconsistent metrics, delayed times to publication and do not measure all factors related to accessibility. This study seeks to capture this additional information and determine its importance for informing accessibility and care coordination.

**Methods:**

The 2018 National Survey for Substance Abuse and Treatment Services (N-SSATS) data were used to identify behavioral health facilities in Indiana and gather baseline information. A telephone survey was administered to facilities with questions parallel to the N-SSATS and additional questions regarding capacity and patient intake. Quantitative analysis includes chi-square tests. A standard qualitative analysis was used for theming answers to open-ended questions.

**Results:**

About 20% of behavioral health facilities responded to the study survey, and non-response bias was identified by geographic region. Among respondents, statistically significant differences were found in several questions asked in both the study survey and N-SSATS. Data gathered from the additional questions revealed many facilities to have wait times to intake longer than 2 weeks, inconsistency in intake assessment tools used, limited capacity for walk-ins and numerous requirements for engaging in treatment.

**Conclusion:**

Despite the low response rate to this study survey, results demonstrate that multiple factors not currently captured in public data sources can influence coordination of care. The questions included in this study survey could serve as a framework for routinely gathering these data and can facilitate efforts for successful coordination of care and clinical decision-making.

## Introduction

Monitoring the successes and challenges of accessing treatment, especially for substance use disorder (SUD), requires accurate, comprehensive and timely information that can support quality improvement efforts and effective coordination of care [1–3]. There are several public sources of data that are maintained by the Substance Abuse and Mental Health Services Administration (SAMHSA) which provide information regarding SUD treatment services and patient utilization [4]. These public databases have varying purposes and are used for both research and clinical decision-making.

These database systems are embedded in health information systems to capture patient-related information. The Treatment Episode Data Set (TEDS) serves as a source for analyzing utilization of treatment in relation to population demographics [5]. The Drug Abuse Warning Network (DAWN) has been used for assessing SUD prevalence and potential demand for treatment [6], though there is potential for underestimation [7, 8]. Information on the availability of treatment services, which is found in the National Survey for Substance abuse Treatment Services (N-SSATS), is used for making referrals, as well as assessments of treatment capacity for SUD in context of policies and population needs [9, 10]. There is rich information in these sources, but differing reporting requirements introduces the potential for variability in estimates and administrative strain on the reporting facilities [11–13].

Variability in metrics may not be the only concern for these public databases. Typically, these sources can have delays of up to a year before publishing reports, increasing the likelihood that data will not accurately represent behavioral health facility characteristics by the time of public consumption [13, 14]. Incorrect or potentially outdated information would undoubtedly have a negative impact on the ability of providers and patients to find appropriate SUD treatment. Additionally, current data does not capture information provider shortages and wait times for intake, two important factors which impact treatment accessibility and utilization [15]. Considering these limitations, the authors of this study developed and administered a survey for the purpose of collecting these additional data elements. The survey provides a potential framework for capturing these and other data elements used to assess facility and workforce capacity.

## Methods

### Data collection

Development of the study survey first began with inclusion of questions from the N-SSATS [16] which would be used for comparison purposes. Additional questions regarding facility workforce capacity, hours of operation and intake procedures were designed and included. The design of these questions was determined by the study research team, which is comprised of clinicians, academic researchers and statisticians. To demonstrate the supplemental information collected, a comparative survey inventory for both the N-SSATS and the study survey is provided in Table 1. The final study survey consisted of five sections: 1. Practice hours and insurance; 2. New Patient intake procedures; 3. Treatment services; 4. Licensed behavioral health professionals; 5. Unlicensed behavioral health professionals. After finalization, the survey was converted into an electronic form using the Research Electronic Data Capture (REDCap™), an online data collection and data management application.

**Table 1:** Comparative survey inventory

Survey Item	N-SSATS	Study Survey
Facility Location	X	X
Operating Agency	X	―
Practice Setting Type	X	X
Practice Hours	―	X
Insurance Accepted	X	X
Payment Methods Accepted	X	X
Substance abuse treatment offered	X	X
Age Groups Served	X	―
Special population groups served (e.g., persons who have experienced abuse, pregnant women, persons with HIV)	X	―
Services for men and/or women	X	―
Comprehensive mental health assessment	X	X
Comprehensive substance abuse assessment	X	X
Continuing Care/Discharge Planning	X	X
Case management	X	X
Substance Detoxification	X	X
MAT Treatment	X	X
Non-substance abuse addiction disorder treatment	X	―
Alcohol Detoxification	X	X
Health Screening	X	X
Assistance with obtaining social services	X	X
Counseling services offered	X	X
Languages spoken at facility	X	X
Health Education Services	X	X
Treatment Programs offered	X	―
Inpatient or Outpatient Programs	X	X
Facility License/Cerficiation/Accreditation	X	―
Funding or Grants	X	―
Patient Referrals Accepted	―	X
Walk-ins accepted	―	X
Appointment required	―	X
Possible wait time	―	X
Smoking permissions	―	X
Patient requirements for treatment	―	X
Types of licensed professionals practicing at facility	―	X
Capacity of licensed professionals	―	X
Types of unlicensed professionals working at facility (e.g., Peer Support)	X	X
Capacity of unlicensed professionals	―	X

X = Captured in survey; ― = Not captured in Survey

Table 1 provides the descriptive a comparative item inventory for the N-SSATS and the study survey. The first column provides the list of items that are considered. The second column provides the indicator for items included in the N-SSATS (X = Included; ― = Not included). The third column provides indicators for the items included in the study survey

N-SSATS data collected in 2018 for behavioral health facilities in Indiana were downloaded from the SAMHSA Behavioral Health Treatment Services Locator, which can be accessed at https://findtreatment.samhsa.gov/. Aside from information on facility name, address and contact information, N-SSATS service data are collected primarily through yes/no or checkbox questions. Therefore, each item is formatted as indicator variables so that if a respondent replied “Yes” or checked the item, a “1” is placed in the data field. These formatted data were uploaded to the electronic survey in REDCap™ to serve as baseline data.

The telephone study survey was administered by trained research staff, who adhered to a survey script and data entry instructions. Though SAMHSA surveys are completed through both paper survey and electronic survey, this study only used the telephone survey to ensure consistency in the method for data collection and entry. Facilities that declined to respond to the survey or did not answer after three phone calls were considered non-respondents. Facilities which answered questions to at least one section of the survey were considered respondents. Survey administration began in August 2018 and concluded in November 2018.

### Descriptive analysis

All facility data gathered in REDCap™ were exported to Microsoft Excel. Differences between respondents and non-respondents with regards in questions asked in the N-SSATS were analyzed using the chi-square analysis to test for non-response bias. A second chi-square analysis was conducted to determine differences in responses to the N-SSATS and the study survey among respondents. Descriptive summary was produced for additional quantitative data collected regarding wait times and provider capacity. Statistical analysis was conducted in SAS 9.4 with significance at α = 0.05. Geographic maps were developed to display the distribution of behavioral health facilities in Indiana using ArcGIS 10.8. The geolocation of behavioral health facilities was based on the geocoordinates provided in the N-SSATS.

### Qualitative analysis

The study survey included three open-ended questions regarding referral, intake and treatment: “What is the intake process for referrals?”; “How do patients access services?”;” Are there requirements for patients to engage in treatment?”. Answers to these questions were summarized into themes after two phases of analysis using the code-to-theory method [17]. In the first phase of analysis, a small team of researchers assigned one or more categories to responses based on level of detail given. Themes were then created by grouping related categories. After presenting the initial themes to the full research team, recommendations were provided for refining categories and themes. Themes and categories were then finalized in the second phase.

## Results

### Descriptive analysis

**Fig. 1:**
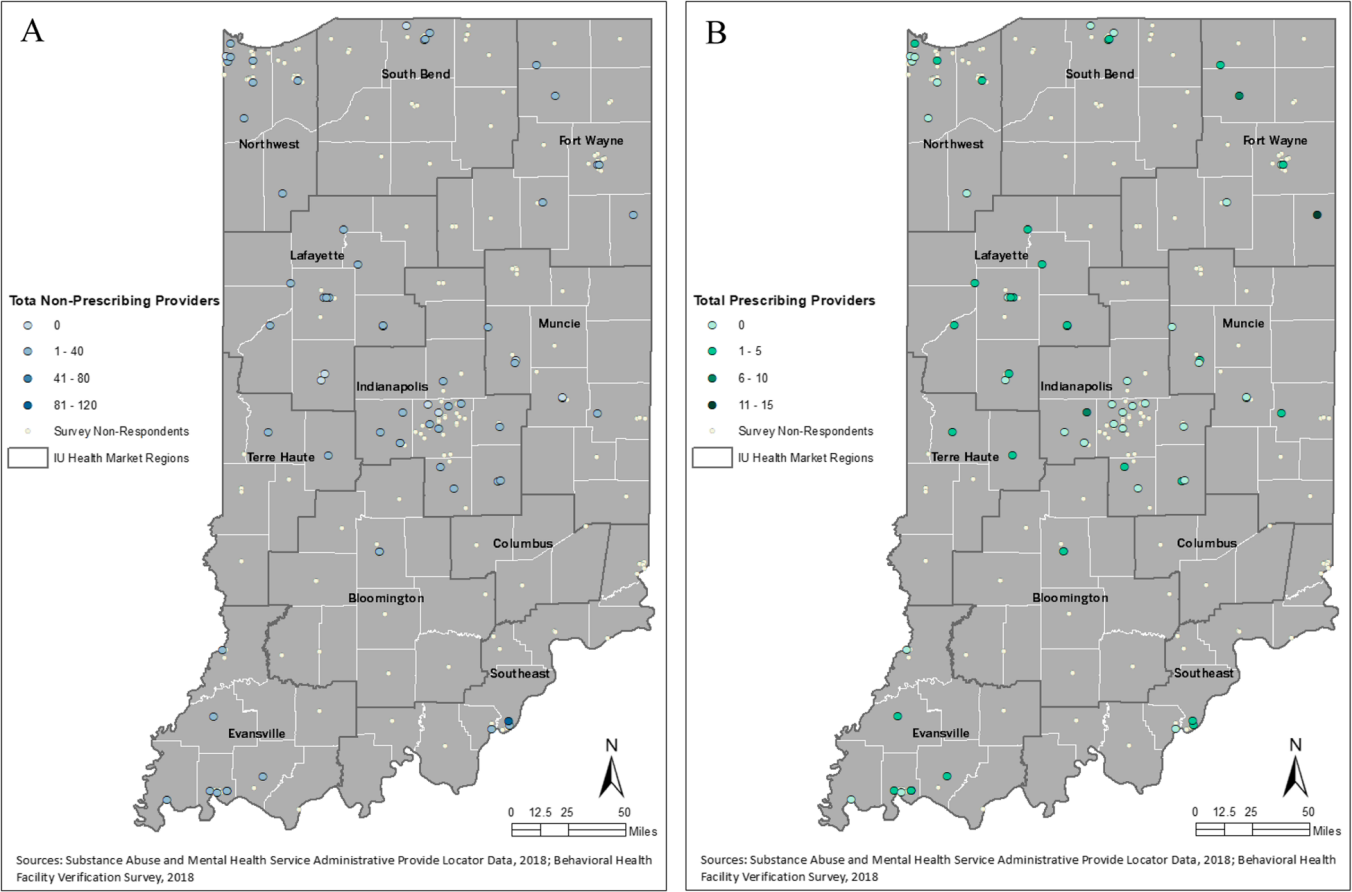
Geographic distribution of respondent and non-respondent behavioral health treatment facilities in Indiana. **a** displays reported number of non-prescribing providers, and **b** displays the reported number of prescribing providers

In the 2018 N-SSATS data, there were 287 SAMHSA-certified behavioral health treatment facilities located in Indiana and included in this study. Among these, 63 (22.0%) behavioral health facilities distributed across 33 Indiana counties responded to the survey. Table 2 provides a summary of the chi-square test for non-response bias based on responses to the 2018 N-SSATS data. Statistically significant differences were found with regards to providing residential services (*p* = 0.009), offering medication-assisted treatment (MAT) health screening (*p* = 0.0173) and regional location (*p* = 0.0064). Geographically, the highest proportion of respondents were in the Evansville, Lafayette, and Indianapolis regions (Fig. 1).

**Table 2:** Difference in N-SSATS data based on study response status

	Respondent		Non-Respondents		p-value
	N	%	N	%	
**Total**	63		224		
**Outpatient Services**					0.7384
Yes	59	93.7	207	92.4	
No	4	6.4	17	7.6	
**Hospital Setting**					0.153
Yes	2	3.2	205	91.5	
No	61	96.8	19	8.5	
**Residential**					0.009
Yes	6	9.5	4	1.8	
No	57	90.5	220	98.2	
**Medication Management**					0.0524
Yes	5	7.9	42	18.8	
No	58	92.1	182	81.3	
**MAT Services**					0.0173
Yes	13	20.6	82	36.6	
No	50	79.4	142	63.4	
**Screening Services**					0.803
Yes	48	67.2	174	77.7	
No	15	23.8	50	22.3	
**Rurality**					0.3945
Urban	53	79.1	163	72.8	
Rurality	15	22.1	61	27.2	
**IU Health Market Region**					0.0064
Bloomington	2	2.9	21	9.4	
Columbus	0	0	4	1.8	
Evansville	9	13.2	14	6.3	
Fort Wayne	6	8.8	25	11.2	
Indianapolis	16	23.5	43	19.2	
Lafayette	11	16.2	8	3.6	
Muncie	6	8.8	27	12.1	
Northwest	9	13.2	27	12.1	
South Bend	4	5.9	27	12.1	
Southeast	3	4.4	20	8.9	
Terre Haute	2	2.9	8	3.6	

Table 2 provides a summary of the chi-square test for non-response bias based on responses to the N-SSATS. Only N-SSATS data are used in this analysis. The first column outlines the variables included in the chi-square analysis. The second column provides the count and distribution of study survey respondent characteristics, and the third column provides the count and distribution of the non-respondents to the study survey. The last column provides the p-value from the chi-square analysis of each variable. Fisher’s exact test was used in instances in which counts for a specific crosstab was less than 5

A second chi-square analysis tested the difference in source data among survey respondents (Table 3). As compared to responses to the N-SSATS data, respondents to the study survey were more likely to report prescribing buprenorphine (17.5% vs. 4.8%; *p* = 0.0233), offering health education (74.6% bs. 19.1%; *p* < 0.0001) and offering a sliding fee schedule (82.5% vs 42.9%; p < 0.0001). Facilities were also less likely to report offering group counseling (47.6% vs 95.2%; p < 0.0001), offering health screening (38.1% vs. 79.2%; p < 0.0001), and accepting self-pay (74.6% vs 96.8%; *p* = 0.0006).

**Table 3:** Chi-square test for differences in responses among study survey respodents

	N-SSATS		Study Survey		p-value
	N	%	N	%	
**Treatment Services**					
Substance Abuse Treatment*					0.0595
Yes	63	100.0%	59	93.7%	
No	0	0.0%	4	6.3%	
Provides Outpatient MAT Treatment					0.8232
Yes	13	20.6%	12	19.1%	
No	50	79.4%	51	80.9%	
Medication Management for MAT					0.0679
Yes	5	7.9%	12	19.1%	
No	58	92.1%	51	80.9%	
Case Management					0.4375
Yes	46	73.0%	42	66.7%	
No	17	27.0%	21	33.3%	
Prescribes or Administers Buprenorphine					0.0233
Yes	3	4.8%	11	17.5%	
No	60	95.2%	52	82.5%	
Group Counseling*					< 0.0001
Yes	60	95.2%	30	47.6%	
No	3	4.8%	33	52.4%	
**Supplemental Services**					
Health Screening					< 0.0001
Yes	48	79.2%	24	38.1%	
No	15	23.8%	39	61.9%	
Health Education					< 0.0001
Yes	12	19.1%	47	74.6%	
No	51	80.9%	16	25.4%	
Aftercare/Continuing Care					0.3914
Yes	51	81.0%	47	74.6%	
No	12	19.0%	16	25.4%	
**Payment Methods Accepted**					
**Medicaid**					0.4592
Yes	38	60.3%	42	66.7%	
No	25	39.7%	21	33.3%	
**Private Insurance**					1.000
Yes	48	76.2%	48	76.2%	
No	15	23.8%	15	23.8%	
**Sliding Fee Scale**					< 0.0001
Yes	27	42.9%	52	82.5%	
No	36	57.1%	11	17.5%	
**Self-Pay**					0.0006
Yes	61	96.8%	47	74.6%	
No	2	3.2%	16	25.4%	

* Indicates where *p*-value for the Fisher Exact Test was used due to one or more classes having less than 5 observations

Table 3 provides a summary of the chi-square test for differences in responses to parallel questions included in the N-SSATS and the study survey among study survey respondents. The first column outlines the variables included in the chi-square analysis. The second column provides the counts and distribution of respondent answers to the N-SSATS questions, and the third column provides the counts and the distribution of respondent answers to the study survey. The last column provides the *p*-value from the chi-square analysis. Fisher’s exact test was used in instances in which counts for a specific crosstab was less than 5

Additional information collected from the study survey is also summarized in Table 4. Over 40% of respondents use a wait list for intake of new patients for SUD treatment, and nearly two thirds (65.4%) of these respondents reported having a wait time greater than 2 weeks. Just under half of respondents (49.2%) reported having at least one provider at the facility with a scope that includes prescribing medications, such as psychiatrists, psychologists, physician assistants and psychiatric advanced practiced registered nurses. Nearly all (90.5%) of respondents reported their location having at least one non-prescribing provider, such as registered nurses and behavioral health counselors and social workers [18]. The geographic distribution of the reported number of providers is presented in Fig. 1. The largest proportion of respondents (30.2%) reported that their clinical professionals work an average of 25–32 h per week, or the equivalent of 3 to 4 days.

**Table 4:** Workforce Capacity Questions

	Number	Percent
**Wait List at facility**		
Yes	29	42.6
No	39	57.4
**Wait Time (*n*** **= 29)**		
Less than 2 weeks	10	34.5
2–4 weeks	15	51.7
5–8 weeks	2	6.9
More than 8 weeks	1	3.4
Unanswered	1	3.4
**Total number of prescribing professionals at facility**^a^		
0	9	14.3
1 or more	31	49.2
No Response	23	36.5
**Total number of non-prescribing professionals at facility**^b^		
0	1	1.6
1 or more	57	90.5
No Response	5	7.9
**Reported average hours per week of licensed professionals in direct patient care**		
No hours in patient care	1	1.2
1–8 h per week	1	1.2
9–16 h per week	10	11.6
17–24 h per week	6	7.0
25–32 h per week	26	30.2
33 or more hours per week	13	15.1
Missing	12	14.0

^a^Includes psychiatrists, psychologists, physician assistants and psychiatric advanced practice registered nurses

^b^Includes registered nurses, addiction counselors, clinical addiction counselors, social workers, clinical social workers, marriage and family therapists and mental health counselors

Table 4 provides a summary of the additional questions asked in the study survey. The first column outlines variables and associated categories. The second column provides the counts of responses in each category, and the third column provides the distribution of responses. A footnote is included which defines what is considered a prescribing provider and non-prescribing provider in this study

### Qualitative analysis

Of the 63 survey respondents, 54 (79.4%) answered at least one of the three open-ended questions regarding intake and requirements for SUD treatment (see Tables 5, 6 and 7).

**Table 5:** Themes and categories identified for the question “What is the intake process for referrals?”

Theme	Categories	N	Description(s)
Overall Intake Process	Assessment and Treatment	16	“Following intake assessment, the patient’s level of care is discussed and assigned.”
	Assessment and Referral	1	Patients are interviewed for intake, then referred to a clinician who creates a treatment plan.
	Treatment by referral only (assessment conducted at a previous site)	2	“This location is typically a continuation of care…”
Intake Assessments	Formal Assessment Process with Standardized Tool	7	American Society of Addiction Medicine (ASAM) Tool; Substance Abuse Subtle Screening Inventory (SASSI)
	General Assessment with Specific Tool or Process (Informal)	9	Pre-screening; psychosocial assessment; depression and anxiety screening
	General Assessment – Non-Specific	27	“An assessment is completed”
Shared Decision Making	Patient involved in decision making	6	“talk about alternatives, look at outpatient vs. inpatient needs”; “patient can decline higher level of care”
	No reference to patients in decision making	43	“will determine if appropriate for this program”
Personnel Involved	Specific personnel/staff referenced	7	Counselor conducting assessment; referral to clinician; substance use coordinator
	No reference to personnel staff	42	

Tables 5 provide the summary of the qualitative analysis for the open-ended questions “What is the intake process for referrals?”, “How do patients access services?”, and “Are there requirements for patients to engage in treatment?”. The first column provides the list of the themes identified, the second column provides the list of categories which fall under each theme, and the third column provides the count of study survey respondents who fall into each category. The last column providers

**Table 6:** Themes and categories identified for the question “How do patients access services?”

Theme	Categories	N	Example(s)
Referral Pattern	Patient Initiated Only	32	“patients have to contact the facility”; “patients can call ahead or walk in”;
	Referral Site Initiated Only	2	“referral comes from court”; “someone from 415 Mulberry location has to initialized referral to this facility”
	Both Patient and Referral Site can Initiate	20	“The facility accepts calls from patients…or outside facilities”; “Referrals from employers, educational facilities, walk ins and court-ordered”
Facility Restrictions	No walk-in accepted	4	“walk-ins are not accepted”; “no walk-ins”
	Limited Availability for Walk-Ins	7	“Walk-in evaluations are reserved for Tuesdays”; “welcome to walk-in though they cannot be guaranteed to be seen”
	No restrictions indicated	13	
Referral Source	Health Care Facility	4	“Someone from 415 Mulberry”; “Providers will call”; “Many come from Fairbanks main hospital”
	Government Agency	8	Court-ordered; Department of Child Services; Recovery Works
	Non-traditional Referral Sites	1	“employers, educational facilities”

Tables 6 provide the summary of the qualitative analysis for the open-ended questions “What is the intake process for referrals?”, “How do patients access services?”, and “Are there requirements for patients to engage in treatment?”. The first column provides the list of the themes identified, the second column provides the list of categories which fall under each theme, and the third column provides the count of study survey respondents who fall into each category. The last column providers

**Table 7:** Themes and categories for the question “Are there requirements for patients to engage in treatment?”

Theme	Categories	N	Example(s)
Level of requirement enforcement	No requirements	7	“no requirements”
	Recommendations for Treatment	3	“no but recommended”; “recommended to attend”
	Specific Requirements Indicated	26	“group therapy”; “Attendance policy”; “support group”
	Based on Treatment (Individualized)	15	“Treatment plans vary”; “individualized”
Counseling requirements	Group Therapy/Support Group	25	“support group is an expectation”; “required group meetings”; “1 h of group therapy a week”
	Counseling	1	“required counseling services off site”
	Family Involvement in Therapy/Counseling	2	“family members are invited”;
	Therapy Frequency	13	“1 h…a week”; “3 days a week”
Adherence Requirements	Medication Requirements	2	“have to be able to take own medications”; “must show up daily for methadone”
	Personal Development	5	“must come in sober”; “clients are required to work on all areas of their life”; “report from patients”; “obtain sponsor within 2 weeks”
	General Attendance Policies	12	“Have to be in before curfew”; “Attendance policy requires that patients attend their sessions”
	Adhere to specific treatment program	2	“Yes, if in substance abuse program”; “Residential program”

Tables 7 provide the summary of the qualitative analysis for the open-ended questions “What is the intake process for referrals?”, “How do patients access services?”, and “Are there requirements for patients to engage in treatment?”. The first column provides the list of the themes identified, the second column provides the list of categories which fall under each theme, and the third column provides the count of study survey respondents who fall into each category. The last column providers

### What is the intake process for patients referred to your facility?

#### Theme 1: overall intake process

Intake process typically involves three phases: 1) coordinating initial appointment/admissions; 2) completing assessments; and 3) determining treatment plans at one location. Responses revealed variation in the way in which the intake process is implemented. There were 16 facilities which indicated following the typical intake process of completing both the intake assessment and treatment plan at the initial appointment. Two facilities indicated continuation of care as their primary service, meaning that completion of an assessment and treatment plan establishment are completed by a referring agency. One facility indicated that their intake process involved completing an intake assessment at the initial appointment, followed by referral to a clinician for treatment planning as part of a separate appointment.

#### Theme 2: intake assessment

A total of 43 respondents indicated that some form of assessment was administered at intake in order to determine the appropriate treatment for the new patient. The most common responses referred to administering a non-specific intake assessment (*n* = 27) (example: “An assessment is completed”). Among those that reported using a formal assessment tool, the American Society of Addiction Medicine (ASAM) tool or the Substance Abuse Subtle Screening Inventory (SASSI) tool were most frequently cited.

#### Theme 3: patient engagement

Six facilities indicated that patients are engaged in shared decision-making regarding treatment during the intake process.

#### Theme 4: personnel involved

Seven facilities identified the clinical personnel that were involved in the intake and assessment process. The specific types of clinical personnel reported to be involved in these processes include clinical counselors, general clinicians, or substance use coordinators.

### How do patients access services?

#### Theme 1: referral pattern

All 54 facilities indicated the method by which a referral to their outpatient clinical treatment can be made. The most common method was a self-referral by the patient (*n* = 32). However, 20 additional facilities indicated that both a patient and a referral site can initiate contact with a treatment facility. For the remaining facilities, referrals were accepted as mandated by a state agency or through continuation of care.

#### Theme 2: facility restrictions

A small number of facilities (*n* = 11) indicated restrictions to referrals. For instance, four facilities indicated that walk-ins were not accepted. The remaining facilities indicated limited availability for walk-ins, such as having designated walk-in days.

#### Theme 3: referral source

Thirteen facilities indicated the specific source from which their referrals are typically accepted. For instance, four identified a specific health care facility as the sources for their referrals, and eight indicated accepting referrals from government agencies, such as the Department of Child Services or the Indiana Recovery Works residential program. One facility reported receiving referrals from non-traditional sites, such as employers or educational institutions.

### Are there requirements for patients to engage in treatment?

#### Theme 1: level of requirement enforcement

When asked whether there were any patient requirements for treatment, 4 requirement levels were identified. These ranged from no patient requirements (*n* = 7) to having individualized requirements (*n* = 15). The predominant response (*n* = 26) was that facilities had requirements specific to the treatment they offered, such as attendance policies for counseling or group therapy.

#### Theme 2: counseling services requirements

Many facilities offered different forms of counseling (*n* = 41) and had requirements based upon the type of counseling offered. For instance, 25 facilities had requirements for attending group therapy or support groups. Another 13 indicated the specific frequency of weekly counseling attendance that was required.

#### Theme 3: additional requirements

Beyond counseling, facilities also reported having other adherence requirements for patients. These included medication management, sobriety and curfew. Other requirements depended on the type of treatment program assigned to the patient.

## Discussion

Real-time behavioral health facility data were collected by this study’s telephone survey to address factors impacting access to care. The N-SSATS collects information that is valuable to informing referrals for behavioral health treatment. This study survey, however, went beyond N-SSATS and gathered information which may have implications for coordination of care and can also help inform potential gaps in accessibility. For instance, results demonstrate that there may be a prevalence of wait lists for intake appointments and limited acceptance for walk-ins among treatment facilities in Indiana. These findings are important when considering that increased delays to treatment initiation are associated with continued substance use and lower likelihood of completing treatment programs [19, 20]. Patients may also experience discouragement with multiple attempts to schedule the first appointment [21].

Delays in the time to initiating treatment have been a persistent issue which disproportionately affects racial minorities, low-income populations, those in the criminal justice system, and those seeking methadone treatment [19, 22]. Barriers to timely admission to a treatment program also impact health outcomes and the quality of services received [23]. Of course, there are many factors which can influence this phase of intake, such as the patient’s insurance status, the type of treatment that is being sought and the referral source, but the impact of these barriers is not independent from each other [23]. For instance, expansion of Medicaid through the Patient Protection and Affordable Care Act has had no significant impact on behavioral health treatment utilization, indicating that coverage alone will not increase access without successful coordination of care [24, 25]. The implementation of quality improvement strategies, such as using electronic health records and national databases to measure wait times and personnel capacity, may assist behavioral health facilities in responding to increasing population demand [3].

In addition to addressing the referral phase of the intake process, results from the qualitative analysis suggest that the use of assessment tools for diagnosing patients and determining treatment plans lacks consistency. The type of assessment tools used ranged from standard national tools to general psychometric tools. Though the type of tool used is at the discretion of the facility, there are concerns with making informed decisions given the differing psychometrics, focus, and target population of many assessment tools [26]. There have been recommendations promoting the use of standard assessment tools such as the SASSI and ASAM [27, 28], but these tools have not proven to have comparable effectiveness [29–34]. Given this evidence, it may be worth examining alternative standard guidelines for using assessment tools which may aid in ensuring patients receive equitable and timely services [35].

Finally, the number of respondents unable to answer or provide a complete assessment of the licensed professionals at their facility is concerning for understanding facility capacity. As with behavioral health treatment and utilization data, there are public sources of workforce data which could be used for verification of workforce capacity. However, many sources lack validity, reliability, and alignment with a minimum data set (MDS) structure [36]. This MDS survey tool design has been used for developments of a state license survey in Indiana which collects supplemental information on professional practice information, capacity, and services provided by each professional [37–40]. Thus, state level workforce data could serve as a supplement to calculating behavioral health service capacity within treatment facilities.

### Limitations

There are notable limitations to this study. First, this study relied on data collected from a survey which may introduce the potential for response bias. This may be reflected by the fact that there was no control as to who at the behavioral health care facility answered the phone and responded to the survey. However, because a publicly available phone number provided in the N-SSATS was used, responses most likely mirror the responses that potential patients or providers would receive when seeking specialized treatment for SUD. Second, the questions designed for collecting additional data from behavioral health facilities were not validated and could have impacted the types of answers provided by behavioral health facilities.

The chi-square test results suggest the possibility of non-response bias based on certain facility characteristics. Additionally, because of the relatively small sample of behavioral health facilities in the state that responded to the survey, the generalizability of the results is limited. Finally, results from the qualitative analyses may be subject to preconceptions; however, the potential for this risk was minimized through the implementation of a standard methodology for qualitative research.

## Conclusions

The results of this study highlight the need for comprehensive and timely information about treatment facilities which can be used for clinical decision-making, facilitating care coordination and assessing organizational effectiveness. Data not typically captured by public sources, such as wait times, intake procedures and current capacity, were helpful for identifying factors which can inform referrals to behavioral health treatment. These factors can also aid in the examination of disparities in the quality and accessibility of treatment services. The data that can be collected from a survey such as this study survey, or from existing data sources, could also be used to support development of referral networks. However, additional research is needed to determine the minimum dataset that is needed to address these issues related to accessibility.

## Data Availability

The datasets used and/or analyzed during the current study are available from the corresponding author on reasonable request.

## Abbreviations

SUD: Substance Use Disorder
N-SSATS: National Survey of Substance Abuse Treatment Services
TEDS: Treatment Episode Data Set
DAWN: Drug Abuse Warning Network
N-MHSS: National Mental Health Services Survey
SAMHSA: Substance Abuse and Mental Health Services Administration
REDCap™: Research Electronic Data Capture
ASAM: American Society of Addiction Medicine
SASSI: Substance Abuse Subtle Screening Inventory
HRSA: Health Resources and Services Administration
